# The role of mental health symptomology and quality of life in predicting referrals to special child and adolescent mental health services

**DOI:** 10.1186/s12888-021-03364-2

**Published:** 2021-07-23

**Authors:** Yeosun Yoon, Jessica Deighton, Alice Wickersham, Julian Edbrooke-Childs, David Osborn, Essi Viding, Johnny Downs

**Affiliations:** 1 EBPU (Evidence Based Practice Unit), UCL and Anna Freud Centre, London, UK; 2 Department of Psychological Medicine, Institute of Psychiatry, Psychology & Neuroscience, King’s College London, London, UK; 3 Division of Psychiatry, University College London, London, UK; 4 Psychology and Language Sciences, University College London, London, UK; 5 Department of Child and Adolescent Psychiatry, Institute of Psychiatry, Psychology & Neuroscience, King’s College London; and South London and Maudsley NHS Foundation Trust, London, UK

**Keywords:** Data linkage, CAMHS referral, Quality of life, Mental health symptoms

## Abstract

**Background:**

Children and adolescents’ mental health problems have been largely assessed with conventional symptom scales, for example, Strengths and Difficulties Questionnaire (SDQ) given that it is one of the mostly widely used measures in specialist Child and Adolescent Mental Health Services (CAMHS). However, this emphasis on symptom scales might have missed some important features of the mental health challenges that children and young people experience including day to day functioning and life satisfaction aspect (i.e. qualify of life).

**Method:**

The study examined longitudinal association between a young person’s self-perceptions of quality of life and mental health difficulties and referral to specialist CAMHS service using a population cohort study (Targeted Mental Health in Schools service data) nested within a large-scale linkage between school (National Pupil Data base) and child mental health service administrative data (South London and Maudsley NHS Foundation Trust children and adolescent mental health services health records). Cox proportional hazard regression to estimate crude and adjusted hazard ratios (HRs) for the association between participant psychopathology, and incidence of CAMHS referral.

**Results:**

Pupils experiencing more behavioural difficulties, had an increased incidence of CAMHS referral (adjusted hazard ratio 1.1, 95% confidence interval 1.0–1.2). However, pupils who reported higher health related quality of life had a lower incidence of CAMHS referral over the follow-up period (adjusted hazard hario 0.94, 95% confidence interval 0.9–0.98).

**Conclusion:**

Children and young people’s perception of their quality of life should be considered at the stages of a clinical needs assessment.

**Supplementary Information:**

The online version contains supplementary material available at 10.1186/s12888-021-03364-2.

## Background

Mental health disorders occur in one in eight children and young people [1]. The most common problems are internalising disorders such as anxiety and depression, with rates highest in females aged between 16 and 19 [1]. At present, only one in four young people with difficulties within a clinical threshold will seek support from specialist Child and Adolescent Mental Health Services (CAMHS) [1]. Furthermore, of those young people and families who are referred to CAMHS, one in four were not accepted into treatment [2]. Demand outstrips supply to such an extent that some CAMHS services set their acceptance thresholds based on imminent risk to self or others, leaving many young people with treatable mental health conditions unable to access CAMHS or facing extra-ordinary waiting times [3].

Past research has focused on barriers to CAMHS access, which include demographic factors, including ethnicity, and indicators of social vulnerability, for example social care or youth justice service contact [4–6]. Peer, family and self-stigma, limited contact with adult caregivers who recognise mental need [7] and a lack of awareness to navigate the supportive services [8], are also known to prevent young people from seeking appropriate help. As these findings suggest, drivers of help-seeking do not just appear to relate to mental health symptom severity but also other contextual burdens that a young person is exposed to.

It is also possible that not all children, with mental health symptoms, who meet clinical disorders thresholds, have unmet needs. Those with a sufficiently positive quality of life – those with good home, school, social lives and a sense of autonomy - may experience much lower impact from their symptoms [9]. For example, some children and young people may be able to manage their mental health challenges and maintain quality of life either through self-management or support from family or other supportive adults/peers, charities and online tools [1, 10].

At present, children and adolescent’s mental health problems have been largely examined using conventional symptom scales, for example Strengths and Difficulties Questionnaire (SDQ) as it is one of the most widely used measures in CAMHS [11, 12]. However, this emphasis on symptom scales has missed some important features of the mental health challenges that lead to contact with mental health services. In particular, less consideration has been given to the impact of mental health problems on day to day function and young people’s ability to derive meaning and enjoyment from life [13].

Furthermore, there has been little work conducted on how quality of life, especially young people’s self-rated quality of life, may relate to help seeking or the recognition of need by adult caregivers. A few longitudinal studies have examined mental health symptoms and environmental variables as predictors of adolescent rated quality of life, showing an expected association between both poorer family function, higher symptom severity and lower quality of life [14, 15]. However, as far we are aware, no studies have examined the longitudinal effect of quality of life on access to CAMHS that the conventional symptom measures may not capture, alongside baseline mental health symptoms and whilst taking into account other potential confounders including education attainment, school environment and family characteristics.

We aimed to address this limitation within this study. To do this, we used a population cohort study nested within a large-scale linkage between school and child mental health service administrative data. Using a historical cohort design, we examined whether a young person’s perceived qualify of life (QoL) alongside their symptoms of emotional and behavioural difficulties was predictive of later contact with CAMHS. We set out to estimate the strength of this relationship, whilst taking into account other potential confounders for CAMHS referral including ethnic, social-economic, social and educational factors.

## Methods

### Data source and study design

This study utilised linked data extracted from three different data sources: TaMHS (Targeted Mental Health in Schools), CRIS (Clinical Records Interactive Search system) and the National Pupil Database (NPD), a routine dataset collated by the Department for Education in England containing information about children and young people’s education, skills and also some social care services data.

Launched in 2008, the TaMHS programme was developed to improve the psychological wellbeing and mental health of children, young people and their families across 71 different local authorities [16]. As part of a national evaluation of the project, online mental health self-report surveys were completed by young people and their families between 2008 and 2010, including 2500 primary and secondary school children from the Southwark and Lambeth boroughs [16].

TaMHS has previously undergone a data linkage with the National Pupil Database (NPD). The NPD contains routinely-collected educational data for all pupils in England’s state schools [17] and was linked to TaMHS via anonymised Pupil Matching Reference (PMR) identifiers [18]. The NPD has, in turn, previously been linked to pseudonymised electronic health records for a cohort of children and adolescents who referred to South London and Maudsley NHS Foundation Trust (SLaM) CAMHS [19, 20]. SLaM CAMHS serves several London boroughs including Southwark and Lambeth, and linkage to the NPD was undertaken for children and adolescents who were referred to SLaM CAMHS in the years 2007 to 2013, thereby capturing the same region and observation period for which TaMHS questionnaire data are available. Thus, a sample of population study from TaMHS and routine health service data from SLaM CAMHS were subsequently available to be linked through previously undergone data linkage work with the NPD.

**Fig. 1:**
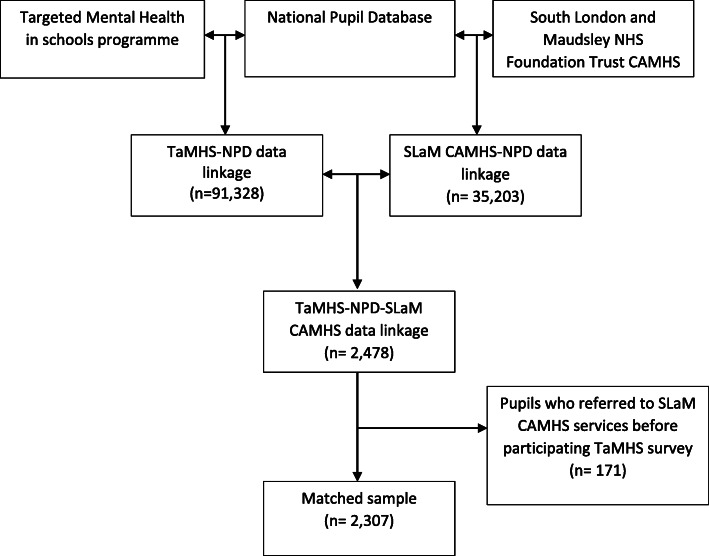
Data linkages used in this study

Therefore, using the PMR identifiers common to all three data sources, we conducted an individual-level data linkage between TaMHS questionnaire data, NPD educational records, and SLaM CAMHS electronic health records for a sample of children and adolescents in Southwark and Lambeth (Fig. 1). From the date that young people participated TaMHS survey for the first time,^1^ this study follows 3-year observation period and tracks a possible CAMHS contact. From this, the study aims to ascertain in a general population sample, what baseline factors are associated with time to a CAMHS referral. We excluded children and adolescents who reached their 18th birthday during the 3-year observation period, who were not resident of the catchment area served by SLaM CAMHS during the TaMHS observation period, or who were referred to SLaM CAMHS services before participating in TaMHS questionnaires. The resulting cohort of *n* = 2307 children and adolescents formed the sample for this study.

### Main outcome

The main outcome of interest was referral to SLaM CAMHS during the 3-year observation period. Successful linkage between TaMHS and SLaM CAMHS electronic health records via the PMR identifier was used as an indicator of whether children and adolescents in the TaMHS cohort had been referred to CAMHS services over a three-year follow-up period. When pupils were referred to CAMHS more than once, we looked at their first referral.

### Main exposures

The main exposure variables were child quality of life and mental health status, as measured by TaMHS questionnaires. This included self-reported emotional and behavioural problems measured with Me and My Feelings^2^ (M&MF) [22], and health-related quality of life (HRQoL) measured with KIDSCREEN-10 [24]. In the analysis, we used total scores of these variables. The detail information about the me and my feeling questionnaire and methods for generating the subscale scoring can be found in supplement document.

### Covariates

A range of potential confounders were extracted at baseline including socio-demographic characteristics and educational factors. Age at baseline questionnaire completion, gender, ethnicity, Income Deprivation Affecting Children Index (IDACI) scores, and school climate were obtained from TaMHS questionnaire data. Free School Meal (FSM) eligibility, Special Educational Needs (SEN) status, English as an additional language, and Key Stage 2 (KS2) maths and English attainment scores were obtained from the NPD. KS2 attainment was standardised relative to a local reference population in the SLaM catchment area for each academic year. The resulting ranked z-scores showed a bimodal distribution, so we dichotomised them above and below the 50th percentile.

### Statistical analyses

All analyses were conducted using STATA (Version 15). Descriptive data were summarised with frequencies and percentages for categorical variables, and means and standard deviations for continuous variables. Chi-squared tests and independent t-tests were used to compare exposure variables and covariates between those who received a referral to SLaM CAMHS in the observation period to those who did not receive a referral. We used Cox proportional hazard regression to estimate crude and adjusted hazard ratios (HRs) for the association between participant psychopathology, covariates (gender, ethnicity, age, FSM and SEN status, KS2 English and math attainment), and incidence of CAMHS referral. The first model examined the crude association between mental health difficulties, health-related quality of life, school climate, and referral to CAMHS. In the second model, sociodemographic characteristics (i.e., age, gender, ethnicity, neighbourhood deprivation index, eligibility of FSM, SEN status and English as additional language) were included. In the third model, educational factors including KS2 attainment and school climate were added.

### Sensitivity analyses

To investigate the potential effect of dichotomising KS2 attainment variables, sensitivity analyses were conducted. We re-ran the analysis either entering KS2 attainment z-scores as a continuous variable, or as a categorical variable denoting quartiles.

## Results

### Socio-demographic, educational and mental health and wellbeing characteristics of the sample

We identified that 2307 children and young people, aged 8 and 13 years at the participation of TaMHS survey were matched through the CRIS-NPD-TaMHS linkage. Those children had no contact with CAMHS previously. During the three-year follow-up period, 106 (4.6%) were referred to CAMHS. Table 1 provides further information on the socio-demographic, educational, mental health and wellbeing characteristics of the children and adolescents, broken down by whether or not they received a SLaM CAMHS referral in the observation period. Pupils who were from more deprived backgrounds (based on FSM eligibility and IDACI score quartiles) as well as those with special educational needs had a higher incidence of referral to SLaM CAMHS (*p* < 0.05). Also, children and young people who were referred to SLaM CAMHS had lower HRQoL scores and higher mental health difficulties scores at baseline (*p* < 0.01).

**Table 1:** Socio-demographic, educational and mental health and wellbeing characteristics of young people with and without referral to CAMHS service

Sample Characteristic	CAMHS referral		*p*-value*
	No (*n* = 2201)	Yes (=106)	
Sex: female, N (%)	1167 (53.0)	64 (60.4)	0.138
Age at first TaMHS survey participation (SD)	10.1 (1.51)	10.23 (1.57)	0.399
Ethnicity, N (%)			
White	700 (31.8)	37%	0.054
Black	753 (34.2)	43%	
Mixed	214 (9.7)	12%	
Asian	252 (11.5)	< 5%	
Any other group	89 (4.0)	< 5%	
Not stated	193 (8.8)	< 5%	
Neighbourhood characteristics, N (%)			
1st (Least deprived)	563 (25.6)	13 (12.3)	0.001
2nd	553 (25.2)	23(21.7)	
3rd	549 (25.0)	29 (27.4)	
4th (Most deprived)	533 (24.2)	41 (36.7)	
Free School Meal: Yes, N (%)	532 (24.2)	36 (34.0)	0.022
Special Education Needs: Yes, N (%)	518 (23.5)	43(40.32)	< 0.001
English as additional language: Yes, N (%)	806 (36.7)	28 (26.7)	0.036
School attainment			
KS2 English z-score below 50 percentile, N (%)	1421 (64.56)	76 (71.70)	0.102
KS2 English z-score above 50 percentile, N (%)	709 (32.21)	26 (24.53)	
KS2 English missing	71 (3.23)	4 (3.77)	
KS2 Math z-score below 50 percentile, N (%)	1342 (60.97)	76 (71.70)	0.017*
KS2 Math z-score above 50 percentile, N (%)	793 (36.03)	26 (24.63)	
KS2Math missing	66 (3.00)	4 (3.77)	
Quality of life/psychopathology			
Mean score: HRQoL – Kidscreen (SD)	30.92 (4.66)	28.96(5.29)	< 0.001**
Mean score: M&MF emotional difficulties (SD)	6.23 (3.49)	7.26 (3.82)	0.003**
Mean score: M&MF behavioural difficulties (SD)	2.62 (2.38)	3.42 (2.33)	< 0.001**
School Climate (SD)	10.37 (3.09)	9.78 (2.78)	0.051

* In the reference of t-test and chi-square analysis, *P* < .05 = *; *P* < .01 = **

### Cox regression models

Table 2 displays the associations between baseline mental health difficulties, HRQoL, and SLaM CAMHS referral, adjusting a range of potential confounders at baseline. Pupils who reported higher HRQoL had a lower incidence of CAMHS referral over the follow-up period (aHR 0.94, 95% CI 0.9–0.98). Pupils reporting more behavioural difficulties, by contrast, had an increased incidence of CAMHS referral (aHR 1.1, 95% CI 1.0–1.2). These findings remained statistically significant after adjusting for potential confounders including socio-demographic characteristics and educational factors. Neighbourhood deprivation characteristics (4th quartile, aHR 2.38, 95%, CI 1.2–4.71) and SEN status (aHR 1.74, 95%, CI 1.12–2.72) at baseline were also positively associated with being referred to CAMHS.

**Table 2:** Multivariable cox regression analysis of the association between CAMHS referral and children and young people’s self-reported mental health and wellbeing

CAMHS referral	Quality of life / psychopathology and school climate (95% CI)	+ Adjusted for socio-demographic factors H.R. (95% CI)	+ Fully adjusted Model H.R. (95% CI)
Quality of life/psychopathology			
Quality of Life: Kidscreen	0.93 (0.90–0.98)**	0.94 (0.90–0.98)**	0.94 (0.9–0.98)**
M&MF: Emotional problems	1.04 (0.99–1.10)	1.04 (0.98–1.1)	1.03 (0.97–1.1)
M&MF: Behavioural problems	1.09 (1.01–1.17)*	1.07 (0.98–1.17)	1.1 (1.0–1.2)*
Sex: Female		1.37 (0.89–2.11)	1.41 (0.90–2.21)
Age at first TaMHS survey participation		1.06 (0.91–1.23)	1.07 (0.91–1.25)
Ethnicity			
White		Reference	Reference
Black		0.79 (0.50–1.25)	0.79 (0.49–1.24)
Asian		0.38 (0.13–1.14)	0.3 (0.09–1.03)
Mixed		0.84 (0.44–1.63)	0.8 (0.41–1.58)
Any other group		0.46 (0.11–1.98)	0.51 (0.12–2.17)
Not stated		–	-
Neighbourhood characteristics			
1st (Least deprived)		Reference	Reference
2nd		1.77 (0.86–3.61)	1.56 (0.75–3.23)
3rd		2.09 (1.03–4.22)*	2.0 (0.99–4.04)
4th (Most deprived)		2.66 (1.35–5.24)**	2.38 (1.2–4.71)*
SEN: Yes		1.81 (1.20–2.72)**	1.74 (1.12–2.72)*
FSM: Yes		1.35 (0.88–2.05)	1.36 (0.89–2.1)
EAL: Yes		0.74 (0.46–1.19)	0.73 (0.45–1.19)
Educational attainment			
KS2 English z-score below 50 percentile			0.98 (0.59–1.63)
KS2 Math z-score below 50 percentile			1.07 (0.63–1.8)
**School Climate**			1.03 (0.96–1.11)

*P < .05; **P < .01

** *SEN* Special Education Needs, *FSM* Free School Meals, *EAL* English as Additional Language

In the sensitivity analyses, we found no significant changes in the direction and magnitude of the association between children’s mental health and wellbeing with CAMHS referral, and educational attainment factors also remained non-significant. The detail information can be found in the supplementary document.

## Discussion

This study demonstrates that children and young people’s self-perceptions of quality of life and mental health difficulties reported via self-report surveys predict the eventual contact with specialist CAMHS services through a three-year follow-up longitudinal analysis. With data linkage work across two large-scale datasets, one community-based, one from specialist services, this study matched children’s self-report mental health and wellbeing information and their mental health service data in two local authorities in South London. Approximately 5% of children in the sample were referred to CAMHS over a three-year follow-up period. Findings indicated that, children’s perception of quality of life was significantly associated with referral to CAMHS after adjusting for baseline mental health symptoms, potential sociodemographic and educational confounders. Children’s perceived behavioural difficulties but not emotional difficulties were also predictive of referral to CAMHS after controlling for a range of personal characteristics. These findings suggest that children’s perceptions of wellbeing and their mental health within their unique environment reflects the likelihood of being referred to CAMHS for their mental health problems. Particularly, our results highlight that children with lower quality of life had the increased likelihood of referral to CAMHS service. This is, to our knowledge, the first study to assess the association between children’s quality of life dimensions and CAMHS referral.

Health-related quality of life (HRQoL) aims to assess children’s subjective health and wellbeing [24, 25] and discriminant validity has been tested using children and adolescent’ emotional and behavioural problem measures including SDQ [9, 26]. This broader construct might have captured aspects of life satisfaction and functioning, not identified using typical mental health difficulty measures such as Me and My Feeling (M&MF). This finding highlights that perceptions of quality of life – of satisfaction and functioning – might be as important as perceptions of mental health symptoms in determining who is in most need of specialist support [27–29]. However, quality of life measures are not widely used in CAMHS or children and young people’s mental health research, with a recent review of adolescent depression studies finding that such measures were included in only 8% of studies [13].

We found other predictive factors that were significantly associated with CAMHS referral including SEN status and neighbourhood deprivation characteristics. Children with SEN status had a higher likelihood of being referred to CAMHS, which is in line with many existing studies examining the close relationship between children with SEN status and mental health disorder [22, 30, 31]. Children who lived in deprived areas were also more likely to be in contact with CAMHS. Compared with children who lived in the least deprived quartile, those who lived in the most deprived quartile were more than twice as likely to be referred to CAMHS service. Such a finding is consistent with previous studies indicating the links with poverty and children’s poor mental health [32–34]. Emotional problems did not significantly predict referral to CAMHS, and the present research cannot explain reasons behind this. On the one hand, it could be argued that perhaps children and young people with emotional problems were accessing other forms of support in the school or community. On the other hand, emotional difficulties could be less likely to be noticed or recognised [35]. Thus, it is important to consider how to best support children and young people who may be experiencing high levels of hidden distress, especially given the high levels of emotional problems in certain groups (see Introduction). It is also of note that ethnic grouping did not predict referral to CAMHS within this analysis, where other research has noted differences in referral rates for different ethnic groups. The lack of difference here may be due to the inclusion of other associated variables such as the deprivation proxies, or it may be due to the small sample for some ethnic groups.

The main strengths of this study are a creation of linked mental health service and community dataset for research to examine the profile of children with CAMHS referral compared to those without referral using the longitudinal follow-up analysis technique. This is one of the few studies that integrate child population level and mental health clinical information via linking between two different datasets. The study is also one of the first longitudinal studies over a three-year follow-up in population sample which takes into account of number of potential confounders including socio-demographic and educational performance. From this, the study presents novel findings that self-reported quality of life at primary school age may have an independent effect on later demands for clinical support. The immediate policy implications are that quality of life decisions have an important effect in seeking support for clinical services, so instruments that just ascertain the mental health needs, may not capture future demand on local services. These findings from this study can substantially contribute to wider local and national initiatives to identify current mental health treatment gaps and targeting service provision. Furthermore, it and illustrates how data linkage methods can be used to evaluate the potential barriers of CAMHS access where significant mental health needs are recognised.

There are some limitations to the current findings. As mentioned previously, the sample was based on pupils who participated on the TaMHS project between 2008 and 2010 through schools in SLaM area and resided in SLaM catchment area during the follow up period. This means that any children and young people who moved out of the SLaM catchment area during the follow-up period were excluded in the sample. TaMHS was often instituted in areas of significant deprivation and high proportions of FSM [16], meaning the sample might not be considered representative of the rest of UK population. The sample was also extracted from two boroughs in South London which is one of the most ethnically diverse regions in the UK; while this diversity is in some ways a strength, it means the population considered is less comparable other areas in the UK and limits the power of analysis due to the small size of sample. Thus, wider data linkage in other areas outside of South London using large samples should be carried out in future research to fully understand the association between accessing CAMHS and children and young people’s self-reported mental health and wellbeing at a regional/national level. Qualitative approaches are also warranted which may provide explanations of local causality which can explain how lower quality of life effect eventually provoke CAMHS referral or those with emotional problems had less contact with CAMHS.

## Conclusion

In summary, our study demonstrated that there was a high prevalence of contact CAMHS among children who had experienced a low quality of life and/or high behavioural difficulties. While referrals to specialist services and routine outcome monitoring during the specialist contact are likely to take account of young people’s reports of their own psychological distress, including behavioural and emotional difficulties, quality of life is not routinely considered. However, our findings suggest that children’s perception of their quality of life contributes to help-seeking from services and should be considered at the early stages of a clinical needs assessment.

## Supplementary Information

**Additional file 1: Table S1.** Multivariable cox regression analysis with education attainment as a quartile variable. **Table S2.** Multivariable cox regression analysis with education attainment as a continuous variable. **Table S3.** Me and My feelings questionnaire.

## Data Availability

The data that support the findings of this study are available (NPD: Department of Education, SlaM CAMHS data: South London and Maudsley NHS Foundation Trust, TaMHS data: EBPU, UCL and Anna Freud Centre) but restrictions apply to the availability of these data, which were used under license for the current study, and so are not publicly available.

## Footnotes

1 Some pupils participated the survey more than once as part of longitudinal cohort.

2 The response rate of SDQ from TaMHS survey were relatively low (around 50%). Also SDQ questionnaires are advised to be used for children aged 11 years or above [21], while Me and My Feeling questionnaires are developed for measuring wider age of young people including below age 11 and cover both emotional and behavioural difficulties [22, 23]. Thus, we used the Me and My Feeling questionnaire to measure the level of mental health difficulties for this study.
