# Supplementary material for: The role of mental health symptomology and quality of life in predicting referrals to special child and adolescent mental health services
Source: BMC Psychiatry. 2021 Jul 23;21:366. doi: 10.1186/s12888-021-03364-2 (PMC8299665; doi:10.1186/s12888-021-03364-2)
Supplement: Supplementary file 1 — Additional file 1: Table S1. Multivariable cox regression analysis with education attainment as a quartile variable. Table S2. Multivariable cox regression analysis with education attainment as a continuous variable. Table S3. Me and My feelings questionnaire. [file 12888_2021_3364_MOESM1_ESM.docx]

Supplementary document: Sensitivity analysis result

Table 1. Multivariable cox regression analysis with education attainment as a quartile variable

| **CAMHS referral** | **Fully adjusted Model**  **Hazard Ratio (95% CI)** |
| --- | --- |
| Quality of life/psychopathology |  |
| Quality of Life: Kidscreen | **0.94 (0.90-0.98)**** |
| M&MF: Emotional problems | 1.03 (0.97- 1.10) |
| M&MF: Behavioural problems | **1.11 (1.02-1.21)*** |
| School Climate | 1.03 (0.95-1.11) |
| Gender: Female | 1.55 (0.98-2.46) |
| Age at first TaMHS survey participation | 1.02 (0.87-1.2) |
| Ethnicity |  |
| White | Reference |
| Black | 0.77 (0.49-1.23) |
| Asian | 0.30 (0.89-1.03) |
| Mixed | 0.82 (0.42-1.63) |
| Any other group | 0.50 (0.12-2.17) |
| Not stated |  |
| Neighbourhood characteristics |  |
| 1^st^ (Least deprived) | Reference |
| 2^nd^ | 1.59 (0.76-3.3) |
| 3^rd^ | 1.98 (0.99-4.04) |
| 4^th^ (Most deprived) | **2.31 (1.17-4.6)*** |
| SEN: Yes | **2.15 (1.35-3.41)**** |
| FSM: Yes | 1.43 (0.93-2.21) |
| EAL : Yes | 0.73 (0.45-1.19) |
| Educational attainment |  |
| KS2 English z-score 1^st^ | Reference |
| KS2 English z-score 2^nd^ | 1.23 (0.64-2.36) |
| KS2 English z-score 3^rd^ | 0.95 (0.45-2.0) |
| KS2 English z-score 4^th^ | 1.19 (0.56-2.56) |
| KS2 Math z-score 1^st^ | Reference |
| KS2 Math z-score 2^nd^ | 1.76 (0.93-3.34) |
| KS2 Math z-score 3^rd^ | 1.35 (0.66-2.79) |
| KS2 Math z-score 4^th^ | 1.59 (0.73-3.47) |

Table 2. Multivariable cox regression analysis with education attainment as a continuous variable

| **CAMHS referral** | **Fully adjusted Model**  **Hazard Ratio (95% CI)** |
| --- | --- |
| Quality of life/psychopathology |  |
| Quality of Life: Kidscreen | **0.94 (0.90-0.98)**** |
| M&MF: Emotional problems | 1.03 (0.97- 1.10) |
| M&MF: Behavioural problems | **1.1 (1.01-1.2)*** |
| School Climate | 1.02 (0.95-1.11) |
| Gender: Female | 1.44 (0.92-2.26) |
| Age at first TaMHS survey participation | 1.08 (0.93-1.26) |
| Ethnicity |  |
| White | Reference |
| Black | 0.79 (0.5-1.26) |
| Asian | 0.30 (0.87-1.02) |
| Mixed | 0.81 (0.41-1.59) |
| Any other group | 0.50 (0.12-2.14) |
| Not stated |  |
| Neighbourhood characteristics |  |
| 1^st^ (Least deprived) | Reference |
| 2^nd^ | 1.57 (0.76-3.25) |
| 3^rd^ | 2.00 (0.99-4.06) |
| 4^th^ (Most deprived) | **2.38 (1.2-4.73)*** |
| SEN: Yes | **1.83 (1.15-2.9)*** |
| FSM: Yes | 1.38 (0.9-2.21) |
| EAL : Yes | 0.73 (0.45-1.18) |
| Educational attainment |  |
| KS2 English z-score | 1.13 (0.5-2.55) |
| KS2 Math z-score | 1.03 (0.47-2.25) |

Table 3.

Me and My feelings questionnaire

|  | Never | Sometimes | Always |
| --- | --- | --- | --- |
| I feel lonely |  |  |  |
| I cry a lot |  |  |  |
| I am unhappy |  |  |  |
| Nobody likes me |  |  |  |
| I worry a lot |  |  |  |
| I have problems sleeping |  |  |  |
| I wake up in the night |  |  |  |
| I am shy |  |  |  |
| I feel scared |  |  |  |
| I worry when I am at school |  |  |  |
| I get very angry |  |  |  |
| I lose my temper |  |  |  |
| I hit out when I am angry |  |  |  |
| I do things to hurt people |  |  |  |
| I am calm |  |  |  |
| I break thing on purpose |  |  |  |

Scoring

The total score is calculated by adding all the 16 items in the scale, rating responses for this scale are scored as follows: Never = 0, Sometimes = 1, Always = 2. Item 15 (“I am calm”) is reverse scored, as follows: Never = 2, Sometimes = 1, Always = 0

The first 10 items comprise the Emotional Difficulties subscale. Items 11-16 comprise the Behavioural Difficulties subscale. To calculate the subscale scores, add the items belonging to each subscale, e.g. add items 1 to 10 to calculate the Emotional Difficulties subscale score. The total score for the Emotional Difficulties subscale is 20, and is 12 for the Behavioural Difficulties subscale, giving an overall Total score of 32.
